# Nanogels of a Succinylated Glycol Chitosan-Succinyl Prednisolone Conjugate: Release Behavior, Gastrointestinal Distribution, and Systemic Absorption

**DOI:** 10.3390/ijms21072376

**Published:** 2020-03-30

**Authors:** Haiyan Zhou, Yuri Ikeuchi-Takahashi, Yoshiyuki Hattori, Hiraku Onishi

**Affiliations:** Department of Drug Delivery Research, Hoshi University, 2-4-41, Ebara, Shinagawa-ku, Tokyo 142-8501, Japan; zhy00630@gmail.com (H.Z.); y-ikeuchi@hoshi.ac.jp (Y.I.-T.); yhattori@hoshi.ac.jp (Y.H.)

**Keywords:** succinylated glycol chitosan-succinyl prednisolone conjugate, nanogel, gastrointestinal distribution, release behavior, effectiveness, toxic side effect, ulcerative colitis

## Abstract

Recently, the potential of nanoparticles (NPs) in ulcerative colitis (UC) therapy has been increasingly demonstrated. Namely, anionic NPs have been found to be accumulated efficiently to the UC damaged area due to epithelial enhanced permeability and retention (eEPR) effect. Previously, a novel anionic nanogel system (NG(S)) was prepared, and evaluated for the efficacy and toxicity. In the present study, release behaviors and biodistribution were investigated in detail to elucidate the functional mechanisms. Rats with 2,4,6-trinitrobenzene sulfonic acid (TNBS)-induced ulcerative colitis (UC) were used as biomodels. In vitro release was examined with or without the contents of the cecum or distal colon. Gastrointestinal distribution and plasma concentrations were investigated after the intragastric administration of 10 mg prednisolone (PD) eq./kg. At pH 1.2 and 6.8, release behaviors were slow, but controlled. Overall release was not markedly different irrespective of coexistence of intestinal contents. In in vivo studies, a large amount of PD was distributed in the lower parts of the gastrointestinal tract 6 and 12 h after administration with NG(S). PD accumulated well in the colonic parts, and prolonged release was noted. The systemic absorption of PD with NG(S) was hardly found. NG(S) concentrated the drug in the colon and showed controlled release. These behaviors were considered to lead to the previously reported good results, promotion of effectiveness and suppression of toxic side effects.

## 1. Introduction

Although the etiology of ulcerative colitis (UC) remains unclear [1], it is regarded as an autoimmune disease. Additionally, UC is induced by genetic factors or is inherited [2]; however, behavioral and environmental factors also appear to contribute to its onset. UC is a chronic relapsing disease that causes colonic inflammation and complications [3,4,5,6]. It is most commonly diagnosed in patients aged between 15 and 35 years, affects 1–2 million individuals in the United States and 5 million individuals worldwide, and its incidence is increasing in newly industrialized countries [6,7,8]. Clinically, prednisolone (PD), a typical glucocorticoid, is widely used to treat UC [9,10,11]. Its current administration methods include oral as well as rectal dosing [12,13]. The route of rectal dosing requires maintaining a laying position for a long time, and is not practical for the upper ulcerative colon. When PD is administrated orally, large quantities of the compound are absorbed by the upper digestive tract and may cause severe toxic side effects, including weight gain, diabetes, osteoporosis, and glaucoma [14,15,16]. Therefore, in the past few years, drug delivery systems (DDSs) have been attracting increasing attention due to their potential to promote therapeutic efficacy and reduce severe adverse effects. DDSs using biodegradable polymers [17], pH-dependent controlled release polymers [18,19], enzyme-controlled release polymers [19], gastrointestinal pressure-controlled release [20], and prodrug approaches [21] have been examined and shown to efficiently deliver drugs to inflamed mucosal membranes and suppress systemic absorption [22,23].

**Figure 1:**
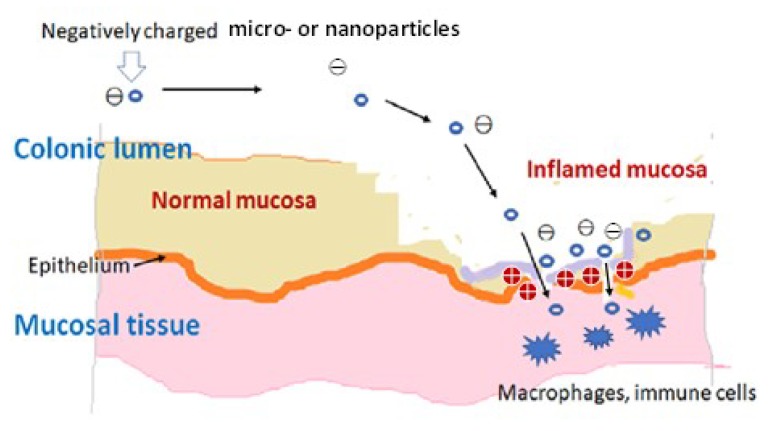
Potential accumulation of nanoparticles (NPs) in intervals of the colonic epithelial cell membrane due to the epithelial enhanced permeability and retention (eEPR) effect.

In recent decades, the potential of nanoparticles (NPs) in UC therapy has been increasingly demonstrated. The inflamed mucosa of UC is characterized by a deficient intestinal cellular membrane, with the loss of barrier function and increased epithelial permeability. Although these structure accounts for only 1% of the surface area of the intestinal mucosa, negatively charged NPs may accumulate easily in damaged areas, including epithelial cells and macrophages (Figure 1), because of an increasing concentration of positively charged proteins around there. Such physiological condition is called the epithelial enhanced permeability and retention (eEPR) effect [24,25]. Pathophysiological studies recently revealed that positively charged particles or gels accumulate in normal and healthy membranes, whereas negatively charged ones may localize in the inflamed colonic mucosa [26,27]. Negatively charged NPs are less likely to cohere with mucoadhesion in the upper gastrointestinal tract, and, thus, may be used to target inflamed regions in the colon.

Previously, we developed the nanogel, NG(S), composed of glycol chitosan-succinyl prednisolone (S-GCh-SP) [26]. NG(S) contains carboxyl esters, and showed gradual release of PD. Such prolonged release by the cleavage of a labile bond such as carboxy esters was attributed to their sensitivities to many elements, including, but not limited to, pH [26,27,28].

Based on these findings, the glycol chitosan (GC)- succinyl prednisolone (SP) conjugate, named GCh-SP, was synthesized, and subsequently modified into a negatively charged conjugate by succinic anhydride (SA), named S-GCh-SP. S-GCh-SP with high drug loading forms a nanogel (NG) due to the interactions of hydrophobic PD moieties and functions as a negatively charged NP because of the presence of succinyl groups in the water-soluble polymer parts [28]. In the previous study, the NG of S-GCh-SP, called NG(S), was examined for preparation procedures and particle characteristics, and evaluated mainly for the efficacy and toxicity. In the present study, effect of intestinal contents on release behaviors, gastrointestinal distribution-time profiles and plasma concentrations were investigated in detail to make clear the functional mechanisms.

## 2. Results and Discussion

### 2.1. Preparation and Characterization of NG(S)

The structure and PD content of S-GCh-SP could be estimated from the ^1^H-NMR data of GCh and S-GCh-SP (supplementary files 1–4). First, as to the structure of original GCh, the substitution degree of glycol groups per chitosan sugar unit was calculated to be 160% (mol/mol), indicating that some glycol groups were combined to C6′-OH and other positions, probably C2′-OH. Additionally, the substitution degree of acetyl groups per chitosan sugar unit was 22% (mol/mol), which was similar to the substitution degree estimated by the supplier. The substitution degree of succinyl groups, calculated from their integrated intensity of 2.2–2.5 ppm, was 85% (mol/mol), in which 28% (mol/mol) was derived from the SP part and 55% (mol/mol) was introduced by the reaction using SA. The PD content, estimated from the integrated intensity of typical PD proton, C1-H and that of sugar protons, was 23.4% (*w*/*w*). The PD content, assessed using UV absorption after ester hydrolysis in 0.1 M NaOH (supplementary file 5), was 20.8% (*w*/*w*; Table 1), and was similar to the content observed from ^1^H-NMR data. Generally, the molar number is not necessarily exactly from the integrated intensity in ^1^H-NMR because that intensity can be affected by the kinds of structures. Therefore, the PD content was determined by the UV absorbance.

Furthermore, the possibility of SA to react with the hydroxy groups of PD and GCh were checked, which was related to analysis of the release mode. According to the reports by Kato et al. [29], the succinylation proceeded preferentially at the amino groups in the present reaction conditions. In addition, when the reactivity of SA to SP was checked in the similar reaction conditions, no succinylation of SP was caused, indicating the hydroxy groups of SP should not be succinylated by SA. Moreover, in the release study from NG(S) in the aqueous media of pH 1.2 and 6.8, only PD was recognized to be released, and ester forms of PD with SA were hardly detected (see Section 2.2. In vitro Release from NG(S)). These supported that only the simple *N*-succinylation of GCh-SP by SA should be caused.

**Figure 2:**
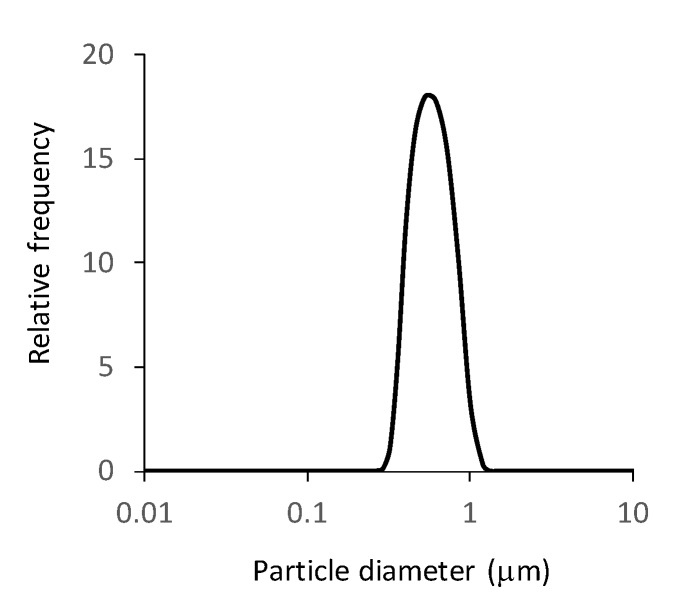
Particle size distribution of NG(S).

The particle characteristics of NG(S) are shown in Table 1. The mean size was 560 nm and mean zeta potential was −34.8 mV. Its size distribution is shown in Figure 2. As to the size distribution of NG(S), the polydiversity index, shown as a value of (size distribution width represented by standard deviation)/(mean size in the distribution) was approximately 0.28. The PD content was 20.8% (*w*/*w*), which was larger than the previous one. The reaction conditions, particularly, pH adjustments in GCh-SP conjugation and reaction with SA appeared to influence the PD content.

Although structure analyses by other measurements were not performed, spectra data such as MS or FT-IR measurement will enable the structural analysis in the form of powder. These data might be needed in the following study.

### 2.2. In Vitro Release from NG(S)

NG(S) samples with and without cecal or distal colonic contents were incubated, and aliquot samples were collected at appropriate time points and analyzed by high-performance liquid chromatography (HPLC). Release profiles were obtained from free PD detected (detection limit = 0.01 mg/mL). Based on a previously reported validation study, free PD evaluated by the direct injection in HPLC reflected that from NG(S) to the outer medium; the free PD concentration obtained using the ultrafiltration membrane (MW cut-off with 30,000) was the same as that of the present method. Therefore, release experiments were conducted in a similar manner.

**Figure 3:**
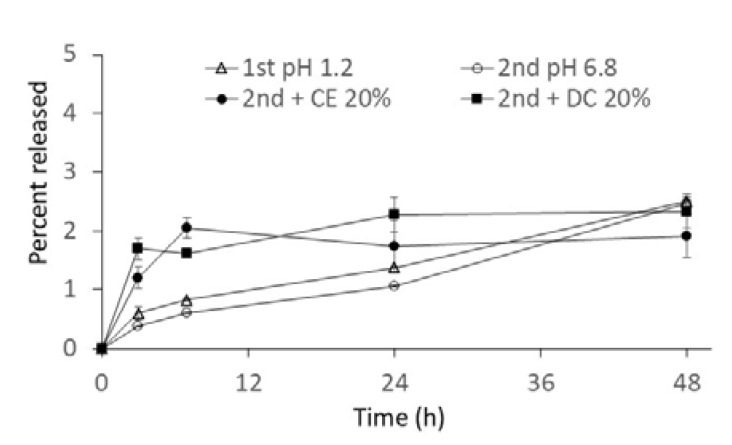
Release profiles from NG(S) with a high drug content (21%) in media of JP 17 first fluid (1st pH 1.2) and JP 17 s fluid (2nd pH 6.8) without or with a 20% cecal content homogenate (2nd + CE 20%) and 20% distal colonic content homogenate (2nd + CD 20%). Results are expressed as the mean ± S.D. (*n* = 3).

In the present study, drug release profiles from NG(S) were investigated in JP 17 first fluid (pH 1.2), JP 17 s fluid (pH 6.8), and a cecal or colonic content suspension (20%, *w*/*v*) in JP 17 second fluid. The first and second fluids are commonly used as simulated gastric and small intestinal media, respectively, and the difference in pH should influence the hydrolysis rate of the ester of the SP moieties in NG(S). Although the medium pH around the diseased area was not known exactly, that was presumed to be weakly acidic to neutral. In this study, the second fluid was used. Release profiles were obtained as shown in Figure 3. Under conditions with no intestinal contents, release was very slow, mean cumulative release ratios gradually increased to approximately 1% and 2% (*w*/*w*) at 24 and 48 h, respectively, and the difference in the release rate between pH 1.2 and 6.8 was small. 

As more SP moieties promote hydrophobic interaction in NG(S), its physical state would be changed. Considering the drug contents, the hydrophobic interaction might increase in the present NG(S) as compared with the previous one (less PD content). On the other hand, the carboxylates of succinyl moieties should cause the repulsion electrically each other and influence the microenvironment of the ester bond. Such change in the physical state might affect the release rate in the present NG(S); more carboxylates around the ester bond might bring about more acidic microenvironment, leading to less hydrolysis rate of the ester.

Moreover, the release rate was affected by the addition of intestinal contents. The initial release rate initially accelerated; however, the effect was small. The slight increase observed in the amount released may have been due to some enzymes already present in the homogenate. However, the macromolecular structures and hydrophobic association of SP moieties give steric hindrance to the enzymes. Therefore, the influence of intestinal contents on release was considered to have been small. Effect of intestinal contents on the hydrolysis of ester prodrugs had been reported before [30,31,32], and intestinal fluid of the cecal and colonic regions appeared to act as the degradation of the ester prodrugs. Although the high molecular weight ester prodrug is not easily degraded by the esterase alone, the ester hydrolysis can be promoted after the degradation of the backbone. Udo et al. reported that the high molecular weight ester could be promoted by the addition of rat cecal contents, which was because the cecal content contained the esterase and enzymes related to the breakdown of sugar backbones [32]. The release of PD from NG(S) was accelerated, though the release extent was small. The initial rapid release might be due to the enzymatic effect by the intestinal contents.

In our previous study, the release rate was dependent on the medium pH. Hydrophilic macromolecules in NG(S) were swollen by immersion in aqueous media, and PD, liberated by ester hydrolysis, was released into the media. In the present study, pH-dependent release was not marked. Furthermore, the release rate was slower than that reported previously [26]. The present NG(S) had a higher content of PD (20.8% (*w*/*w*)) than the previous study (13.7% (*w*/*w*)) [26]. In NG(S) with a high drug content, the hydrophobic interaction of the SP moieties was considered to be strong. In these rigid structures, the ester of the SP moieties may not have been subjected to hydrolysis, leading to the smaller release of free PD. Similar features were reported for the NG with a higher PD content; the chondroitin sulfate-glycyl prednisolone conjugate nanogel (CS-GP-NG), in which CS-GP-NG with a higher PD content showed a smaller release rate and had a weaker influence on the difference caused by medium pH [33]. The difference in PD content of NG(S) appeared to influence the release rate and stability of the ester bond.

In the cecal or colonic content suspension (20%, *v*/*v*) in JP 17 s fluid, only the initial release rate was slightly increased, and the later release became slow. Various components contained in the intestinal contents may interact with the surface molecules of NG(S). Such interaction of the medium component with NPs was reported by Zhang et al. [34]. The modification of NG(S) by the components of the intestinal contents might influence the release patterns. Similar phenomena were reported for chitosan-SP conjugate microspheres [35], the release rate of which in a 20% (*w*/*v*) intestinal suspension had been suppressed to nearly 50% of that in the buffer alone.

Finally, the released compounds were confirmed in order to assure that only PD was released in the media. It was demonstrated in the following experimental results. (1) SP did not react with SA in a similar reaction condition. (2) The PD concentration in the ultrafiltrate was not changed after the further 24 h incubation of the ultrafiltrate. (3) After the ultrafiltrate was treated in 0.1 M NaOH, the PD concentration did not increase. These results supported that only PD was released and other PD esters were not existed.

### 2.3. Drug Distribution in the Gastrointestinal Tract after the Intragastric Administration of NG(S)

**Figure 4:**
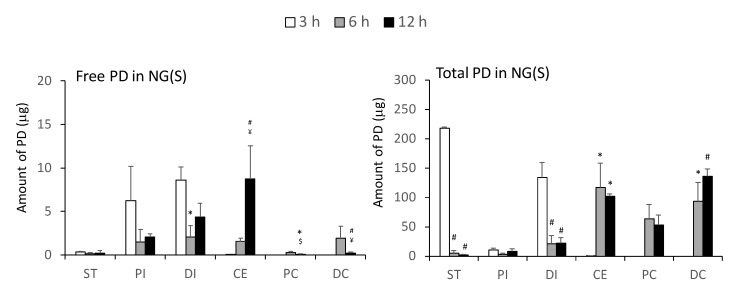
Gastrointestinal distribution of PD after the oral administration of NG(S) and PD alone to rats with TNBS-induced UC at 10 mg PD eq./kg. Results are expressed as the mean ± S.E. (*n* = 4, except for the stomach, 1 h (*n* = 3)). * *p* < 0.05 vs. 3 h; ^#^ *p* < 0.01 vs. 3 h; ^$^ *p* < 0.05 vs. 6 h: ^¥^ *p* < 0.01 vs. 6 h.

The distribution of the drug in the gastrointestinal tract of UC rats was investigated after the intragastric administration of NG(S) and compared with that after dosing with PD alone. The gastrointestinal tract of 2,4,6-trinitrobenzene sulfonic acid (TNBS)-induced UC rats was divided into 6 parts: the stomach (ST), proximal small intestine (PI), distal small intestine (DI), cecum (CE), proximal colon (PC), and distal colon (DC), on which the operation was performed such that the length of PI = the length of DI and the length of DC = 2 × the length of PC. The contents of each part were removed with a spatula, including mucosal surface materials. Free and total (free + conjugated) PD were investigated, and the distribution profiles for NG(S) were shown in Figure 4. Free and total drugs were both observed mainly in the stomach and small intestine at 3 h. Most of PD was detected as the conjugated form, the amount of which was 10-fold or higher than that of free PD. Six and twelve hours after dosing, a large amount of the drug was distributed in the CE, PC, and DC. In these periods, the amount of conjugated PD was 10-fold or higher than that of free PD. These results indicated that NG(S) transfers well to the large intestinal parts containing the colon, and that free PD may be slowly released there. Furthermore, NG(S) distributed in the large intestine appeared to remain there for a long period. These drug distribution patterns suggested that NG(S) efficiently targeted the colonic diseased part. NG(S) distributed in the inflamed area gradually released PD, which may be taken up by inflammatory cells, leading to the suppression of inflammation. The amount of total PD recovered in the gastrointestinal tract was nearly 20% (*w*/*w*) at 3 h, and approximately 15–16% (*w*/*w*) at 6 and 12 h. Low recovery ratios were considered to be attributed to (1) the contents not being completely collected with a spatula, in particular, NG(S), attached to the mucosal surface, potentially remaining there, and (2) the decomposition of PD itself in the intestinal fluids; though it was not checked in detail. Since recovery ratios were similar between 6 and 12 h, NG(S) appeared to be fairly stable in the gastrointestinal tract.

Drug distribution after the dosing of PD alone at 10 mg/kg in a similar UC model has already been reported by Yano et al. [14]; the whole tissue (contents + tissue) was homogenized and the homogenate was used in a distribution study. Drug distribution following the administration of PD alone appeared to be limited to the upper gastrointestinal tract. After 3 h, the majority of PD was recovered in the stomach and small intestinal parts, and the total amount recovered was approximately 20%. Furthermore, PD was not detected 6 or 12 h after dosing. PD orally administered at 5 mg/kg was previously shown to be absorbed well and eliminated quickly from the systemic circulation [35]. Therefore, NG(S) was considered to move more gradually and efficiently to the lower intestine than PD alone, resulting in enhancement of delivery to the diseased area.

### 2.4. Plasma Concentration of PD after the Intragastric Administration of NG(S)

**Figure 5:**
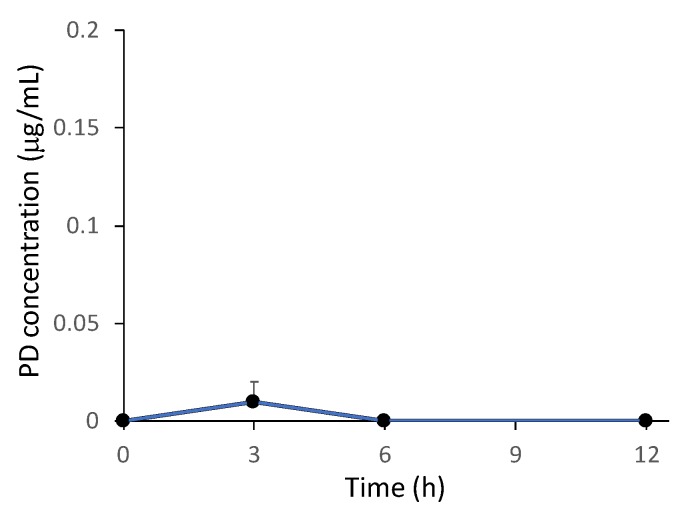
Time course of plasma PD levels after dosing of NG(S). Each point represents the mean ± S.E. of four rats.

Dissolved PD is absorbed well in the upper intestine [35,36,37]. Furthermore, according to the study by Yano et al. [14], PD was quickly absorbed into the systemic circulation after oral dosing with a suspension of PD alone at 10 mg/kg. This good absorption of free PD is associated with side effects [14,34]. Therefore, the plasma PD levels detected were considered to be an indicator of the severity of side effects. In the present study, plasma samples were collected immediately before each excision time point (3, 6, and 12 h) in gastrointestinal distribution studies (Figure 5). A mean plasma level of free PD was detected at approximately 0.01 μg/mL 3 h after dosing. The plasma level of free PD was below the detection limit (0.01 μg/mL) at 6 and 12 h. The negligible appearance of PD in the plasma after the intragastric dosing of NG(S) suggested a decrease in side effects with NG(S).

## 3. Materials and Methods

### 3.1. Materials and Animals

#### 3.1.1. Materials

Glycol chitosan (GCh; MW = ca. 100,000, deacetylation degree = approximately 80% (mol/mol)), PD, SP, SA, and 1-ethyl-3-(3-dimethylaminopropyl) carbodiimide hydrochloride (WSC) were purchased from Wako Pure Chemical Industries, Ltd. (Osaka, Japan). TNBS was purchased from Sigma Chemical Company (St. Louis, MO, USA). Sephadex G50 (fine grade) was obtained from GE Healthcare Bio-Sciences AB (Uppsala, Sweden). Pentobarbital solution, named Somnopentyl, was purchased from Kyoritsu Seiyaku Corporation (Production No. 7231101, Tokyo, Japan). All other chemicals were of reagent grade.

#### 3.1.2. Animals

Male Wistar rats (7 weeks old, 200–210 g) were purchased from Tokyo Laboratory Animal Science Co., Ltd. and raised in a room with a 12-h light-dark cycle. Room temperature and relative humidity were maintained at 23 ± 1 °C and 60% ± 1%, respectively. Animals were fed with the specific breeding diet MF provided by Oriental Yeast Co., Ltd. (Tokyo, Japan) with water available ad libitum. The experimental protocol was approved by the committee on Animal Research of Hoshi University (Tokyo, Japan; project identification code: 26-048, approval date: 20 April 2018) and animal experiments were performed in accordance with the Guiding Principles for the Care and Use of Laboratory Animals of Hoshi University.

### 3.2. Equipment

UV-VIS absorption was measured with a Beckman DU640 spectrophotometer (Beckman Coulter, Inc., Brea, CA, USA). HPLC was used to detect PD with the Shimadzu SPD-10AV VP UV-VIS detector set at a wavelength of 246 nm and Shimadzu C-R7A plus Chromatopac (Kyoto, Japan). The size and ζ-potentials of the particles produced were measured using an ELS-Z2 apparatus (Otsuka Electronic Co., Ltd., Osaka, Japan). ^1^H-NMR spectra were measured with a JNM-ECA600 II spectrometer (JEOL, Tokyo, Japan). 

### 3.3. Preparation of GCh-SP and S-GCh-SP

**Figure 6:**
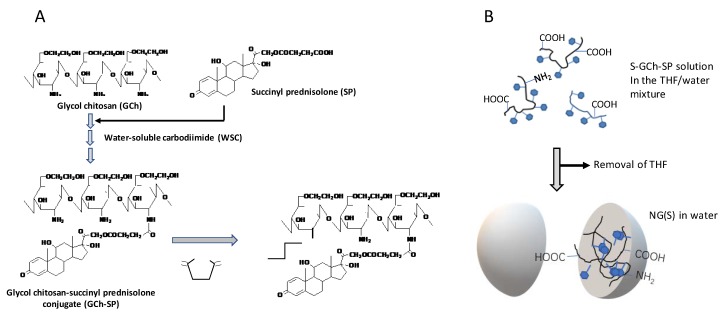
(**A**) Synthetic scheme of GCh-SP and S-GCh-SP. GCh: glycol-chitosan, SP: succinyl prednisolone, GCh-SP: GCh-SP conjugate, S-GCh-SP: succinylated GCh-SP, WSC: water-soluble carbodiimide, SA: succinic anhydride. (**B**) A diagram showing the formation of NG(S) in water.

The preparation scheme of GCh-SP and S-GCh-SP is shown in Figure 6A. These conjugates were produced in a similar manner to that described previously [28]. During the reaction, 1 M HCl and 1 M NaOH were used to adjust pH. GCh (100 mg) was dissolved in 15 mL water, pH was adjusted to 6.2–6.3, and SP (100 mg) was dissolved in 5 mL of tetrahydrofuran (THF). After mixing evenly, the pH of the resulting material was adjusted to approximately 6.3. WSC (500 mg) was gradually added and stirred under ice bath conditions for 1 h, and the reaction was then continued at room temperature (25 °C) for another 23 h. After gel chromatography, macromolecular fractions were collected and dialyzed extensively against water using a cellulose tube. The residue in the tube was obtained as an aqueous suspension of the NG of GCh-SP, named NG(G). In the next step, THF and 0.5 M phosphate buffer (pH 7) were added to aqueous medium containing NG(G) (150 mg) at a ratio of 1/3 (*v*/*v*) and an excessive amount of SA (1050 mg) was then gradually added and stirred for 3 h. Throughout this period, pH and temperature were maintained at approximately 7.0 and 15 °C, respectively. The mixture was subjected to gel chromatography, and high-molecular-weight fractions were collected and dialyzed in a similar method to that for NG(G). The resultant mixture was obtained as an aqueous suspension of a NG of S-GCh-GP, named NG(S) (Figure 6B). 

The structure of S-GCh-SP was confirmed from its ^1^H-NMR spectrum. The mixture of dimethylsulfoxide (DMSO)-d_6_/D_2_O (1:1, *v*/*v*) was used as a solvent. Tetramethylsilane (TMS) was used as a standard (0 ppm) for the proton signal. The PD content of each NG was investigated as follows: NG(G) and NG(S) were heated at 45 °C for 10 min in 0.1 M NaOH aqueous solution. The solutions were then centrifuged. The supernatants were collected and spectrophotometrically measured at 246 nm. The PD content was calculated based on the absorbance of free PD in 0.1 M NaOH aqueous solution.

### 3.4. Production of the UC Model

UC animal models were produced by treating rats with TNBS [23,38,39]. TNBS was dissolved in 50% (*v*/*v*) ethanol aqueous solution at a concentration of 80 mg/mL. After rats were fasted for 2 days, TNBS solution (0.25 mL) was instilled into the colon of each rat, 8 cm from the anus, using a catheter. 

### 3.5. In vitro Release Studies

In vitro release experiments were performed using JP 17 first fluid (pH 1.2), JP 17 s fluid (pH 6.8), and JP 17 second fluid containing 20% (*w*/*v*) cecal or 20% (*w*/*v*) distal colonic contents, in which the distal colon (DC) meant the latter 2/3 part of the whole colon. The NG(S) suspension in JP 17 first or second fluid with no intestinal contents was prepared at a concentration of 40 μg PD eq./mL. Regarding release tests with intestinal contents, incubation samples were prepared as follows. Three days after the instillation of TNBS, rats were sacrificed by injecting excessive Somnopentyl intraperitoneally, and the wet cecum (CE) or DC was collected, weighed, and diluted at 20% using JP 17 second fluid. The diluted contents were further homogenized with a glass homogenizer using a Teflon pestle, and homogenates were filtered with gauze and used to prepare incubation samples. The final incubation sample was set as NG(S) in a cecal or distal colonic suspension (20% (*w*/*v*) wet content) at a concentration of 50 μg PD eq./mL.

Each mixture was incubated by horizontal shaking at 60 rpm at 37 °C. At appropriate time points, a small portion of the suspension was withdrawn from each mixture and processed by the method described below.

Regarding the mixture with no intestinal contents, immediately after each cycle of sampling, 100 µL of 0.1 M acetate buffer (pH 4) was mixed with each sample taken (50 μL) to suppress further release. The resultant mixture was analyzed by HPLC to assess the release of PD. Regarding the incubation with intestinal contents, after each cycle of sampling, 100 µL of water was mixed with each sample taken (100 μL) using a vortex mixer. Methanol (140 μL) and 20% (*w*/*v*) trichloroacetic acid aqueous solution (40 μL) were added separately. The mixture was stirred vigorously with a vortex mixer. After centrifugation at 3000 rpm for 10 min, the supernatant was analyzed by HPLC.

### 3.6. Drug Distribution Studies in the Gastrointestinal Tract after Intragastric Administration

**Figure 7:**
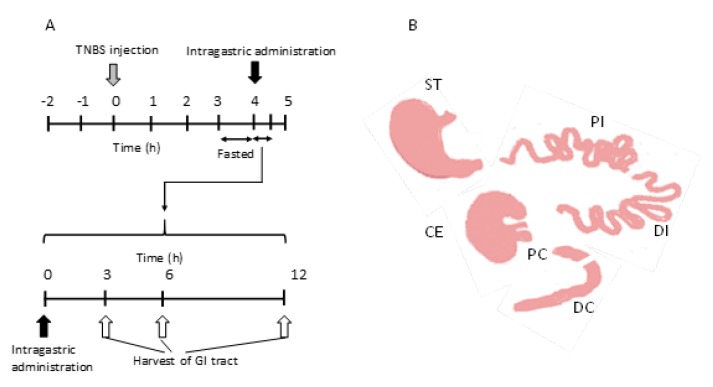
Animal experiment procedure of drug distribution in the gastrointestinal tract of UC model rats. (**A**) After fasting for 48 h, TNBS was injected from the anus, and 3 days later, rats that lost 20% of their body weight were fasted for another 24 h, subjected to intragastric dosing, and sacrificed after 3, 6, and 12 h. (**B**) Diagram of the division of the gastrointestinal tract. ST, stomach; PI, proximal small intestine; DI, distal small intestine; and CE, cecum; PC, proximal colon; DC, DC. The length of PI = the length of DI; The length of DC = 2 × the length of PC.

TNBS-induced UC model rats were prepared as described above. Three days after the instillation of TNBS, twelve rats were divided into three groups (*n* = 4), and fed with water available ad libitum, but fasted for 24 h prior to intragastric administration. Each rat was administrated 1 mL of the NG(S) suspension (equivalent to 10 mg PD/kg). At appropriate intervals (3, 6, and 12 h), rats were sacrificed by injecting excessive Somnopentyl intraperitoneally (Figure 7A). The gastrointestinal tract was cut and divided into six parts: ST, PI (anterior half of the small intestine), DI (posterior half of the small intestine), CE, PC (1/3 front side of the colon), and DC (2/3 rear side of the colon; Figure 7B). The contents in each part were collected by washing with cold PBS (pH 7.4). The contents of ST, CE, and DC were homogenized with a glass homogenizer with a Teflon pestle under ice bath conditions, and diluted to 8 mL with cold PBS (pH 7.4), while the other contents were similarly homogenized, but diluted to 4 mL using cold PBS (pH 7.4).

Free and total (free + conjugated) PD concentrations in the samples collected were assessed as follows. (1) Processing to detect free PD: this was performed in a similar manner to in vitro studies. Water (100 μL) was mixed with each sample collected (100 μL) using a vortex mixer. Methanol (140 μL) and 20% (*w*/*v*) trichloroacetic acid aqueous solution (40 μL) were added separately. The mixture was then stirred vigorously with a vortex mixer. After centrifugation for 10 min at 3000 rpm, the supernatant was analyzed by HPLC. (2) Processing to detect total PD: 50 µL of 0.1 M NaOH was added to 50 µL of the homogenate, followed by an incubation at 45 °C for 10 min. After that, 50 µL of 0.1 M HCl and 50 µL of water were added to the mixture to neutralize alkali, then stirred vigorously with a vortex mixer. Methanol (140 μL) and 20% (*w*/*v*) trichloroacetic acid aqueous solution (40 μL) were added separately. The mixture was stirred vigorously with a vortex mixer. After centrifugation at 3000 rpm for 10 min, the supernatant was analyzed by HPLC.

### 3.7. HPLC Assay

HPLC was performed at room temperature (25 °C) using a Shimadzu LC-6AD pump with a Shimadzu SPD-10AV VP UV-VIS detector set at a wavelength of 246 nm and Shimadzu C-R7A plus Chromatopac (Kyoto, Japan). A YMC Pack ODS-AM column (I.D. of 6 mm, length of 150 mm; YMC Co., Ltd., Kyoto, Japan) was used as the analytical column. A mixture of acetonitrile and 50 mM citrate buffer with pH adjusted to 4.1 with phosphoric acid (35:65, *v*/*v*) was used as the mobile phase. The injection volume was 20 µL, and the flow rate was set at 1 mL/min. PD concentrations were assessed using an absolute calibration curve.

### 3.8. Statistical Analysis

A one-way analysis of variance (ANOVA), followed by Scheffé’s post hoc test, was used to assess the significance of differences. A significant difference was set as *p* < 0.05.

## 4. Conclusions

In the traditional method used to treat severe UC, a typical steroid anti-inflammatory drug, PD is highly effective in clinical practice; however, the long-term use of PD is associated with severely toxic side effects mainly due to its non-specific body distribution, resulting in various hormonal actions. Researchers in various countries are attempting to overcome these issues by suppressing non-specific drug distribution and promoting efficient drug delivery to the target site, leading to the decrease in the amount of drug administered. In recent decades, NPs have been attracting interest in UC therapy. Due to their pathophysiological characteristics, negatively charged NPs specifically accumulate at the diseased site of UC and are phagocytosed by inflammatory cells around these areas. Such NPs, accumulating in damaged membrane and inflammatory cells, can stay long in the damaged region.

In the present study, a negatively charged NG, NG(S), was successfully prepared and its targeted drug delivery profiles, gastrointestinal distribution, plasma concentration, and release behavior were evaluated. The distribution results of NG(S) showed that it moved smoothly to the lower gastrointestinal parts, and remained well in the lower intestine including colon, as compared with PD alone. Plasma concentration profiles showed that PD was hardly absorbed into the systemic circulation after dosing with NG(S). Moreover, NG(S) with a higher PD content (20.8%) exhibited very slow release under acidic and neutral environments. The slower release by the present NG(S) with a PD content of 20.8% might be attributed to its more compact structures formed by the hydrophobic interaction among succinyl prednisolone moieties, resulting in the suppression of accessibility of water-based media. These release characteristics and biodistribution features of NG(S) make it suitable as a DDS for UC therapy.

## Figures and Tables

**Table 1:** Particle characteristics and drug content of NG(S).

Preparation	Particle Size (nm) ^1^	Zeta Potential (mV) ^1^	PD Content (%, *w*/*w*) ^1^
NG(S)	560 ± 41	−34.8 ± 0.3	20.8 ± 0.4

^1^ Results are expressed as the mean ± S.D. (*n* = 3).

## Supplementary Materials

The following are available online at https://www.mdpi.com/1422-0067/21/7/2376/s1.

## Abbreviations

CE	cecum
DMSO	dimethyl sulfoxide
DC	distal colon
DI	distal small intestine
e-EPR	epithelial enhanced permeability and retention
GCh	glycol chitosan
GCh-SP	glycol chitosan-succinyl prednisolone conjugate
NG	Nanogel
NG(G)	glycol chitosan-succinyl prednisolone conjugate nanogel
NG(S)	succinylated glycol chitosan-succinyl prednisolone conjugate nanogel
NP	nanoparticle
PC	proximal colon
PD	prednisolone
PI	proximal small intestine
SA	succinic anhydride
S-GCh-SP	succinylated glycol chitosan-succinyl prednisolone conjugate
SP	succinyl prednisolone
ST	stomach
THF	tetrahyrofuran
TNBS	trinitrobenzene sulfonic acid
UC	ulcerative colitis
WSC	water-soluble carbodiimide
