# Supplementary material for: Nanogels of a Succinylated Glycol Chitosan-Succinyl Prednisolone Conjugate: Release Behavior, Gastrointestinal Distribution, and Systemic Absorption
Source: Int J Mol Sci. 2020 Mar 30;21(7):2376. doi: 10.3390/ijms21072376 (PMC7178247; doi:10.3390/ijms21072376)
Supplement: Supplementary file 1 [file ijms-21-02376-s001.pdf]

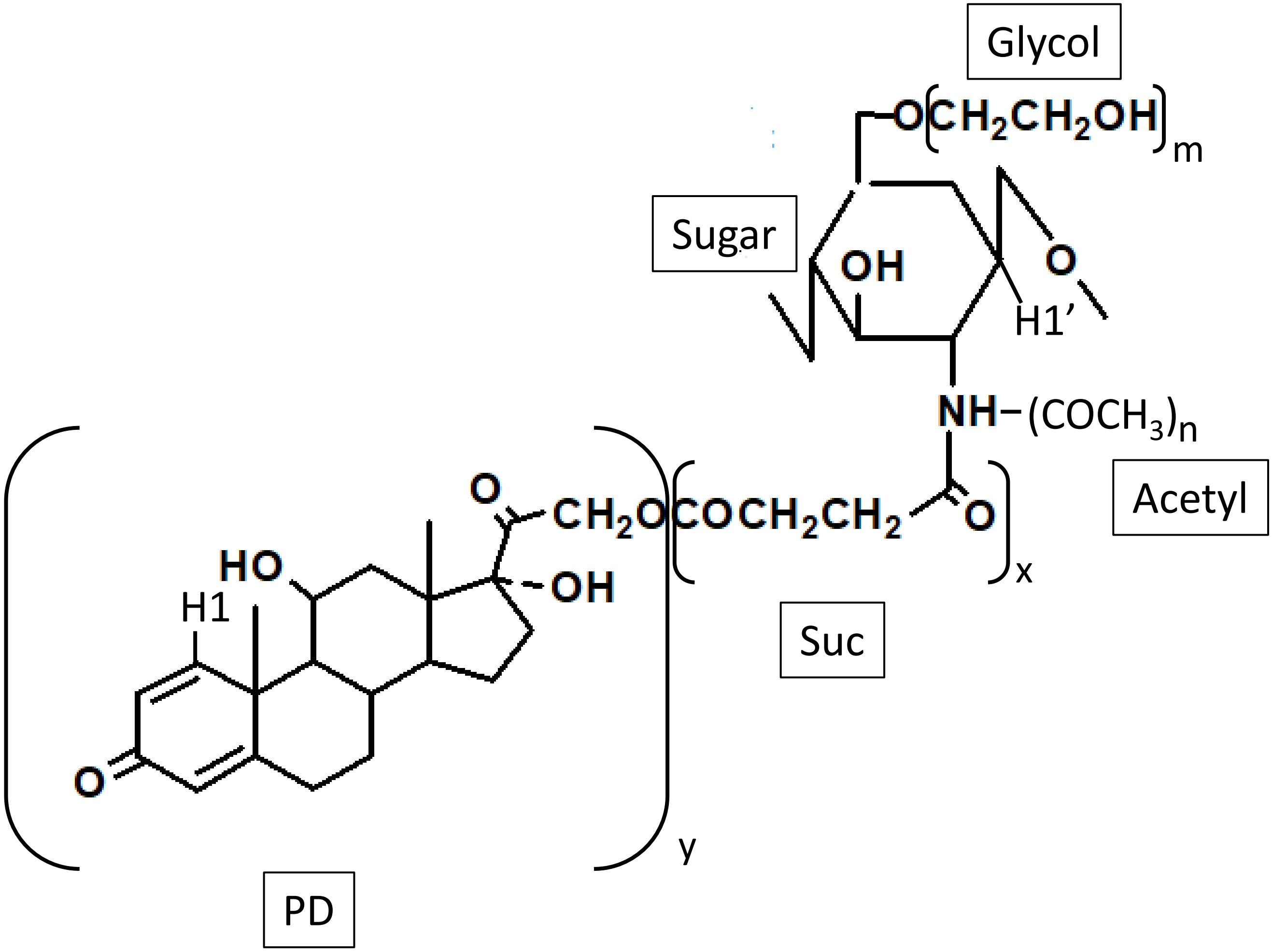

Sapplementary file 1. Proposed structure of S-S-GCh-SP for composition analysis from ^{1}H-NMR.

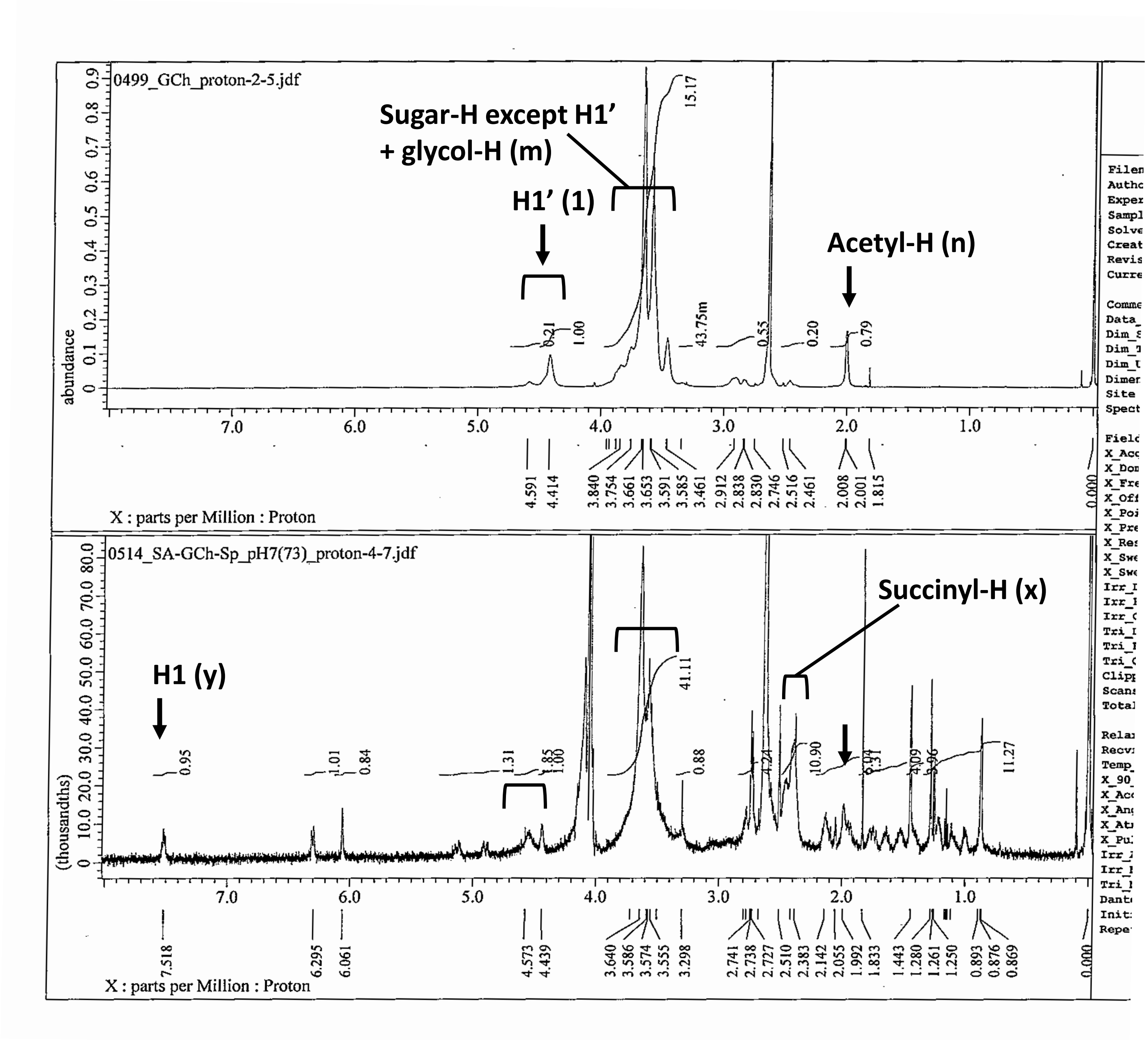

Sapplementary file 2. Assignment of each proton of S-GCh-SP. The character or number in the charts shows a molar number in the structure in suuplementary file 1.

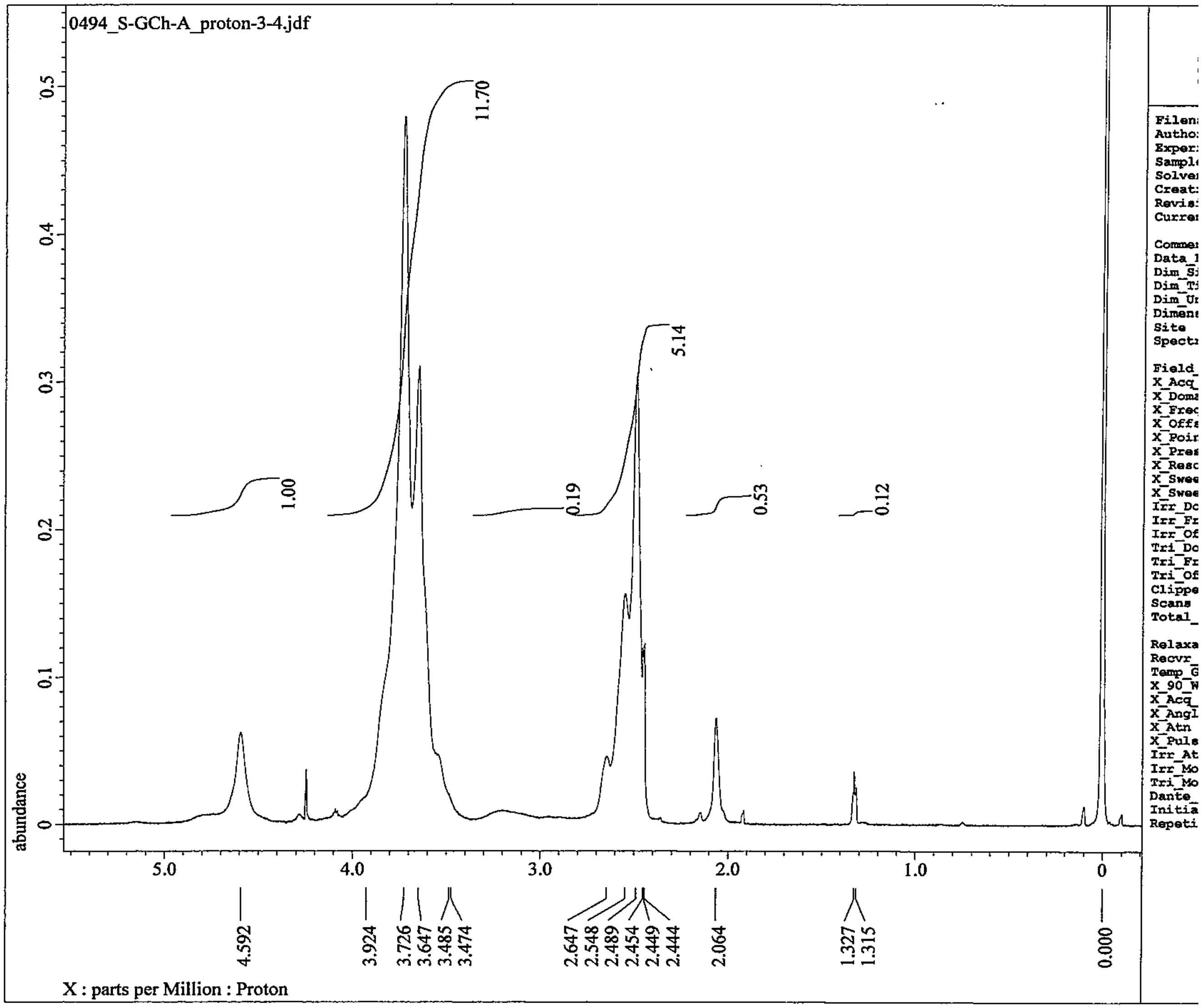

Supplementary file 3. The ^{1}H-NMR spectrum of S-GCh synthesized in the similar reaction.

			cal	obs	normalize(mol)			mol ratio
GCh		H1'	**1**	**1.21**	**1**			
		other H’	6 + 4m	15.17	12.53719008		m=	1.63429752
		COCH3	3n	0.79	0.652892562		n=	0.21763085

			cal	obs	normalize			mol ratio	
S-GCh-SP		H1'	1	1.85	0.564315252				
		other H’	**6 + 4m**	**41.11**	**12.54**		m=	1.635	
		COCH3	3n	1.5	0.457552907		n=	0.15251764	
		Suc	4x	10.9	3.324884456		x=	0.83	
		PD	y	0.95	0.289783508		y=	0.28	
							**x-y =**	**0.55**	**Succinylation degree**

0.28 mol	PD part	amount
PD	MW=360	
**PD content**	**23.42116269**	**(%, w/w)**

Supplementary file 4. Determination of molar ratio of each part (m, n, x, y) and PD content calculated from the 1H-NMR spectra. The m value over 1.0 means glycol group is attached to the parts other C6’ position. The PD content = 23.4 % (w/w).

(A)

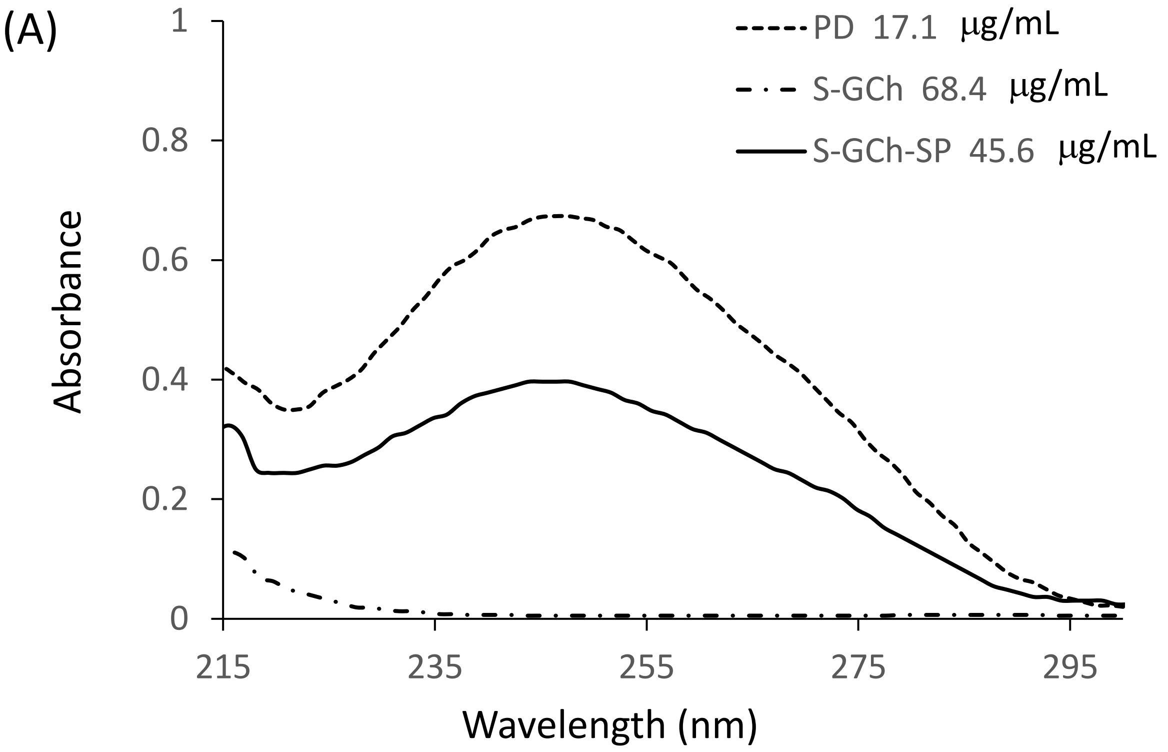

(B)

	mg/mL	conc (ug/mL)	abs(246)
PD	1.71/100	17.1	0.68
S-GCh	1.71/25	68.4	0.01
S-GCh-SP (NG(S))	1.14/25	45.6	0.39
		**PD content =**	**20.9559**

Supplementary file 5. UV absorption profile of PD, S-GCh and S-GCh-SP (treated at 45 oC for 10 min) in 0.1 M NaOH (A), and PD content calculated from the UV absorption at 246 nm. The PD content = 21.0 % (w/w). The PD content was estimated to be 20.8 % (w/w) in triplicated measurements.
